# Functional Analysis of DNMT3A DNA Methyltransferase Mutations Reported in Patients with Acute Myeloid Leukemia

**DOI:** 10.3390/biom10010008

**Published:** 2019-12-18

**Authors:** Daria A. Khrabrova, Andrei G. Loiko, Anastasia A. Tolkacheva, Natalia A. Cherepanova, Maria I. Zvereva, Olga V. Kirsanova, Elizaveta S. Gromova

**Affiliations:** 1 Chemistry Department, Lomonosov Moscow State University, Moscow 119991, Russia; 2 Department of Biochemistry and Molecular Pharmacology, University of Massachusetts Medical School, Worcester, MA 01655, USA

**Keywords:** DNA methyltransferase Dnmt3a, missense mutations, leukemia, DNA methylation, S-adenosyl-l-methionine, DNA-protein binding

## Abstract

In mammals, DNA methylation is necessary for the maintenance of genomic stability, gene expression regulation, and other processes. During malignant diseases progression, changes in both DNA methylation patterns and DNA methyltransferase (MTase) genes are observed. Human de novo MTase DNMT3A is most frequently mutated in acute myeloid leukemia (AML) with a striking prevalence of R882H mutation, which has been extensively studied. Here, we investigate the functional role of the missense mutations (S714C, R635W, R736H, R771L, P777R, and F752V) found in the catalytic domain of DNMT3A in AML patients. These were accordingly mutated in the murine Dnmt3a catalytic domain (S124C, R45W, R146H, R181L, P187R, and F162V) and in addition, one-site CpG-containing DNA substrates were used as a model system. The 3–15-fold decrease (S124C and P187R) or complete loss (F162V, R45W, and R146H) of Dnmt3a-CD methylation activity was observed. Remarkably, Pro 187 and Arg 146 are not located at or near the Dnmt3a functional motives. Regulatory protein Dnmt3L did not enhance the methylation activity of R45W, R146H, P187R, and F162V mutants. The key steps of the Dnmt3a-mediated methylation mechanism, including DNA binding and transient covalent intermediate formation, were examined. There was a complete loss of DNA-binding affinity for R45W located in the AdoMet binding region and for R146H. Dnmt3a mutants studied in vitro suggest functional impairment of DNMT3A during pathogenesis.

## 1. Introduction

DNA methylation is an important epigenetic modification essential for the regulation of cell processes in both eukaryotes and prokaryotes. In eukaryotes, DNA methylation is necessary for the maintenance of genomic stability, gene expression regulation, and other processes [1,2]. DNA methylation is maintained by DNA methyltransferases (MTases), which transfer a methyl group from co-factor S-adenosyl-l-methionine (AdoMet) to the five positions of the cytosine residue mainly within CpG dinucleotides [3]. Changes in the methylation process occur in various human pathologies [4]. During human cancer development, there are alterations in the pattern of methylation and multiple disorders in the MTases, including missense mutations [4,5,6,7]. The function of mammalian Dnmt3a is to methylate DNA de novo [3].

According to the CBioPortal cancer clinical research database, DNMT3A missense mutations account for 1.7% of all known DNMT3A abnormalities (215 studies, 56,243 patients). DNMT3A missense mutations can be divided into two groups according to their localization and different disease types [4]. The first group includes mutations in the regulatory DNMT3A domain, which are most common in the development of overgrowth syndrome with intellectual disability [8,9]. The second group includes mutations in the catalytic domain, which are most characteristic in the development of acute myeloid leukemia (AML) [10,11,12,13,14,15]. AML is the most common leukemia among adults, and the incidence is increasing with aging [13]. Although the main cause of the disease is still unknown, some risk factors have been identified, including mutations in *DNMT3A* gene [14].

### The Preliminary Results

**Figure 1:**
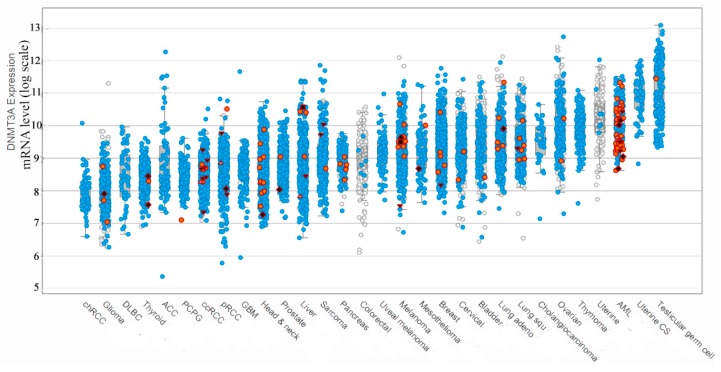
DNMT3A expression during different cancer types among patients (created with CBioPortal tools based on The Cancer Genome Atlas (TCGA) database information). The height of the column corresponds to the change in mRNA production. Red dots mark point mutations, blue dots mark no mutation, white dots mark not sequenced samples, red triangles mark frameshifts and splicing disorders, red squares mark other types of disorders. One dot represents one blood sample.

We have analyzed the DNMT3A expression among the different cancer types (Figure 1) using the information of the recent clinical studies included in The Cancer Genome Atlas (TCGA) [15] and CBioPortal database [16]. Interestingly, the maximum expression of DNMT3A connected to mutated forms of *DNMT3A* was observed only in the case of AML patients with a wide range of levels of mRNA encoding mutated DNMT3A. It should be noted, that in addition to the hyperexpression of the mutated DNMT3A in AML cells, the aberrant methylation pattern also has been reported [17]. The exchange of R882 to histidine (R882H) is the hotspot mutation during AML progression, which occurs in 60% of all mutations [10]. It is the most well studied in terms of influence on human DNMT3A functioning [18]. R882H leads to an 80% loss of DNMT3A activity due to the inability of mutant DNMT3A to form active tetramers with regulatory protein DNMT3L [18]. Notably, other mutations are spread throughout DNMT3A in AML, although these occur at a lower frequency (Table 1 and Figure 1). To our knowledge, there is very little information on how these mutations may impact the DNMT3A function.

For several mutations that are located within the DNMT3A/3A and DNMT3A/3L interaction interfaces, the disruption of the DNMT3A ability to form active tetramers and diverse levels of activity was shown [18,28,29]. In addition, mutations of DNMT3A at its DNA binding residues decrease methylation activity [30].

This study aimed to examine the functional consequences of the widespread AML missense mutations found in the human DNMT3A catalytic domain. Therefore, we studied the murine Dnmt3a catalytic domain, which is highly homologous to its human counterpart. We demonstrated in vitro that substitutions of amino acid residues that do not belong to conserved motifs among MTases had a strong impact on the methylation reaction. This will help to explain the disease phenotypes and changes in the DNA methylation pattern observed in AML patients.

## 2. Materials and Methods

### 2.1. Chemicals, Oligonucleotides, and Enzymes

AdoMet and AdoHcy were from Sigma (St Louis, MO, USA). The buffers used were: A, 20 mM HEPES, pH 7.5, 100 mM KCl, 1 mM EDTA, 1 mM 1,4-dithiothreitol; B, 8 mM phosphate, pH 7.4, 0.15 M NaCl, 3 mM KCl, 0.5% Tween-20. Oligonucleotides (Table 2) were from Syntol (Moscow, Russia) with the fluorescent label 6-carboxyfluorescein covalently attached to the 5′- end phosphate through the aminoalkyl linker -NH-(CH_2_)_6_. Oligonucleotides containing 2-pirimidinone were synthesized by S.N. Mikhailov. Restriction endonuclease Hin6I was from SibEnzyme (Novosibirsk, Russia).

### 2.2. Site-Directed Mutagenesis

Plasmid pET28a(+) carrying the catalytic part of the murine *dnmt3a* gene with the N-terminal His_6_-tag was kindly provided by A. Jeltsch. All amino acid changes were introduced by site-directed mutagenesis. The sequences of all mutated plasmids were checked by Sanger sequencing.

### 2.3. Protein Expression and Purification

Plasmid pET41b carrying the murine *dnmt3l* gene with N-terminal His8-tag and C-terminal GST was kindly provided by G.L. Xu. The C-terminal GST was not removed during further protein purification. All proteins were expressed in *Escherichia coli* BL21(DE3) strain (Stratagene, La Jolla, CA, USA) and purified by metal-affinity chromatography on Co^2+^ Talon^®^ resin (GE Healthcare, Chicago, IL, USA) as previously described [31]. The proteins were analyzed in 12.5% PAG with 0.1% SDS, the purity was >95%. Concentrations of the proteins were determined using the standard Bradford technique.

### 2.4. CD Spectroscopy

Circular dichroism measurements were performed on a Chira Scan spectrophotometer (Applied Photophysics, Surrey, Leatherhead, UK) using a 1-mL cuvette with 0.5-mm path length in 1× buffer A with 100 nM AdoHcy at 15 °C. All protein concentrations were about 0.5 g/L. The baseline was recorded for buffer A in the presence of 100 nM AdoHcy. The spectra for each probe were measured three times and then averaged.

### 2.5. Methylation Assay

WT and mutant Dnmt3a-CD activities were analyzed by the protection of the methylated DNA from cleavage by restriction endonucleases [32]. The 30 bp fCG/GCf DNA substrate with two FAM labels (Table 2), containing the CpG methylation site within G↓CGC Hin6I recognition site (the cleavage position is indicated with an arrow) was used. Three hundred nanomolar fCG/GCf was methylated with 2 μM WT Dnmt3a-CD or mutants in buffer A in the presence of 25 μM AdoMet at 37 °C. The reaction was stopped by heating to 95 °C for 1 min. After methylation, fCG/GCf was digested with Hin6I for 1 h at 37 °C in the same buffer with added Mg^2+^ (3 μM). DNA duplex fCG/GCf cleaved without prior methylation was used as a control. Reaction mixtures were analyzed in 20% PAG with 7 M urea and visualized with a Typhoon FLA 9500 scanner (GE Healthcare, Chicago, Illinois, USA). The fluorescence intensities of intact DNA and cleavage products were determined using GelAnalyzer 2010a software. The extent of DNA cleavage (ω) was calculated as a ratio of fluorescence intensity of the cleaved DNA to the total fluorescence intensity of the intact and cleaved DNA. The extent of methylation (*M*) was calculated using the equation:

(1) $$M=\;\frac{\omega_{0}-\;\omega_{Dnmt3a}}{\omega_{0}}\;\times \;100\%,$$

where ω_0_ and ω_Dnmt3a_ are the extent of DNA cleavage without or after methylation [32].

The concentration of the methylated DNA [(CH_3_)-DNA], was calculated using the equation:

[(CH_3_)-DNA] = *M* × C_DNA_, (2)

where C_DNA_ is the initial DNA duplex concentration.

To obtain time courses of the methylation, the test samples were taken from the reaction mixtures after 1.5, 3, 4.5, 6, 7.5, 9, 15, 20, 40, 60, or 90 min. The initial slopes of the curves were determined by linear regression, and the initial rates of methylation (*v_0_*) were calculated. Along with this, the methylation of the fCG/GCf DNA substrate by WT and mutant Dnmt3a-CD during 2 h was performed, and *M* values were calculated.

In addition, methylation of the fCG/GCf DNA substrate by WT or mutant Dnmt3a-CD in the presence of Dnmt3L during 2 h was determined. Reaction mixtures contained 120 nM fCG/GCf, 1 μM Dnmt3L, 1 μM WT or mutant Dnmt3a-CD, and 2.5 mM AdoMet in buffer A. The MTase and Dnmt3L solutions were preincubated for 30 min at 37 °C, and then the reaction was started by the addition of the other components to these solutions. Further analysis was conducted as described above.

### 2.6. Formation of Covalent Intermediates between Dnmt3a and DNA Duplexes Containing 2-Pyrimidinone

Three hundred micromolar DNA duplexes fCG/GZ and CGZ/GCf were incubated with 6 μM WT Dnmt3a-CD or mutants in buffer A in the presence of 100 μM AdoHcy for 1 h at 4 °C. The reaction mixtures were analyzed in 12% PAG with 0.1% SDS and were visualized as described above.

### 2.7. DNA Binding

WT Dnmt3a-CD or mutants binding to 30 bp fCG/CG DNA substrate (100 nM) with one FAM label was studied using fluorescence polarization (P) in buffer A in the presence of 100 μM AdoHcy. Measurements were performed using a Cary Eclipse spectrofluorometer (Varian, USA) with excitation at 495 nm and emission at 520 nm at 25 °C in 120-µL cuvettes with a 1-cm pathlength, P was defined in terms of the vertical (I_v_) and horizontal (I_h_) emission components as

P = (I_v_ − GI_h_)/(I_v_ + I_h_), (3)

where G is the instrumental correction factor, which was measured once before each experiment. DNA substrate was preincubated with AdoHcy, and fluorescence polarization of free fCG/CG (P_0_) was measured. WT Dnmt3a-CD or mutants were added as 1–2 or 4–5 µL aliquots, respectively, up to a final concentration 1.5–4 μM, and the P value of bound DNA was recorded after 2 min of incubation. Dependences of P values on the total concentration of Dnmt3a-CD were obtained. The dissociation constants (K_d_) for the complexes were calculated by approximation of the obtained dependences using the 3-parameter Hill equation:

(4) $$P\;=\;P_{0}\;+\;\frac{{\left[E\right]}^{n}}{{\left[K_{d}\right]}^{n}+{\left[E\right]}^{n}},$$

where [E] is the Dnmt3a-CD concentration, n is the Hill coefficient.

### 2.8. Western Blotting

Probes containing 0.5 or 1 µg of R181L in buffer A were analyzed in 15% PAG with 10% SDS and were transferred to the membrane using a Trans Blot Turbo Transfer System (Bio-Rad, Hercules, California, USA) according to the instructions of the supplier. The membrane was blocked in milk, washed in buffer B, incubated with 1:5000 mouse HRP monoclonal anti-His_6_-tag antibodies, and then with 1:15000 secondary antibodies. The membrane was scanned at ChemiDoc XRS+ system using the Immune-Star HRP kit (Bio-Rad, Hercules, CA, USA) with a 5 s exposition.

### 2.9. Computational Modeling

DNA-(DNMT3A-CD)-DNMT3L complex structure was modeled using Chimera 1.10.2 software based on PDB file 6F57 [30].

### 2.10. Database Analysis

The following databases describing cancer genetic changes were used to search for mutations in *dnmt3a* gene associated with AML: OncoKB (Precision Oncology Knowledge Base), CBioPortal (The cBioPortal for Cancer Genomics), TCGA (The Cancer Genome Atlas) and COSMIC (Catalogue of Somatic Mutations In Cancer) with selection of AML and default search parameters. The number of patients and COSMIC references along with allele frequency of the mutation were used as criteria for the frequency determination. DNMT3A expression during different cancer types was analyzed with CBioPortal tools based on the TCGA database information with default parameters.

## 3. Results

**Figure 2:**
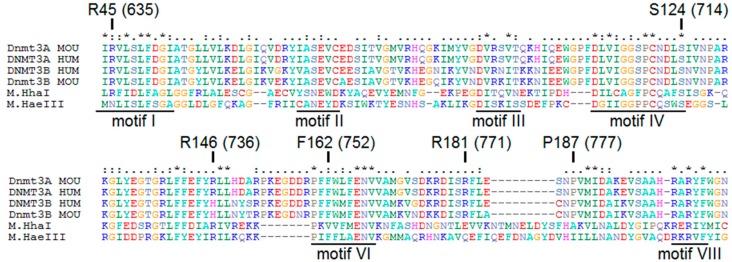
Alignment of the sequences of the catalytic domains of human and murine methyltransferases (MTases) Dnmt3a and Dnmt3b and prokaryotic MTases HhaI and HaeIII with underlined conserved motives (I-X) [33] and location of mutated amino acid residues. The negatively charged amino acids are given in red, the positively charged ones are given in blue, the polar ones are given in violet, the aromatic/non-polar ones are given in green, proline is given in gray, cysteine is given in wine-red, glycine is given in light orange, histidine is given in magenta. Highly conserved amino acids are given stars above, conserved amino acids are given two dots above, often preserved amino acids are given one dot above. The mutated amino acid residues are indicated.

To identify the most common mutations in patients with AML, analysis of the missense mutations’ frequency occurrence in the DNMT3A catalytic domain was performed. It was based on the data [12,19,20,21,22,23,24,25,26,27] extended with recent clinical studies data from cancer databases (Table 1). Mutations in the DNMT3A catalytic domain indicated among a large number of patients (R635W/Q, S714C, and R736H/C/A/P) were found. In addition, mutations of potential interest in terms of properties of the substituted amino acid residues (Figure 2), even though they were not the widespread mutations, were identified (P777R, R771L, and F752V). As a result, six amino acid exchanges S714C, R736H, R771L, R635W, P777R, and F752V corresponding to murine S124C, R146H, R181L, R45W, P187R, and F162V were selected for further research on their impact on Dnmt3a-CD functioning (Figure 2). According to COSMIC, all chosen mutations lead to the development of cancer and practically do not occur in healthy cells. They have not been previously studied in detail in terms of the effect on the murine Dnmt3a-CD.

### 3.1. Dnmt3a-CD Purification

In this study, the murine Dnmt3a-CD was used to evaluate the effects of mutations observed in human DNMT3A. Murine Dnmt3a-CD is easier to purify, has an identical primary structure to the human enzyme, and can function without the regulatory part [3], which makes it an excellent model system to study DNMT3A functioning.

**Figure A1:**
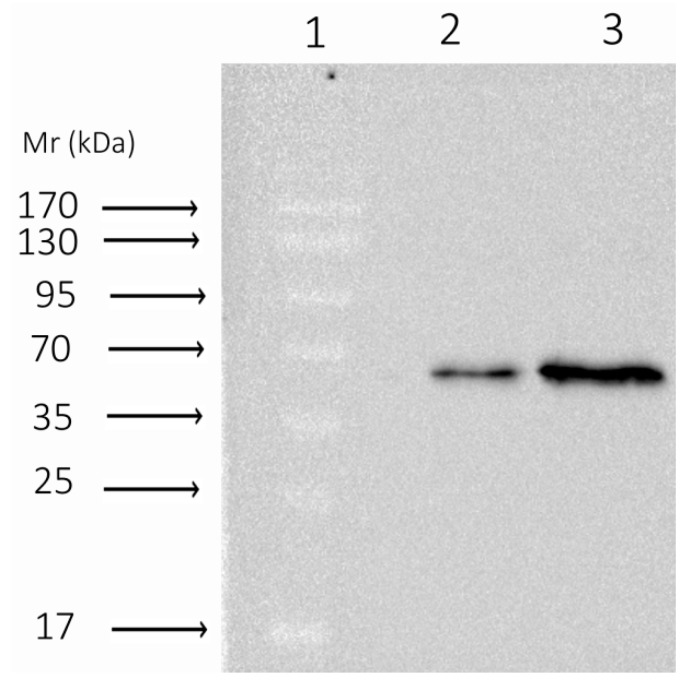
R181L Western blot. Lane 1 represents the protein ladder; Lanes 2 and 3 represent protein dilution 1:10 and 1:15, respectively. Fifteen percent PAG with 10% SDS, 1:5000 mouse HRP monoclonal anti-His_6_-tag antibodies. Molecular mass of the Dnmt3a-CD 37 kDa, the expected second lane of 20 kDa is not observed.

The WT and mutant Dnmt3a-CD were expressed in *E. coli* as C-terminal His_6_-proteins and purified by metal affinity chromatography. The concentration of WT (37 kDa), S124C, and R45W was 12–38 µM. The concentration of mutants P187R and F162V was only 2–3 µM, probably due to their weak expression. In the case of R181L, the second fraction with molecular mass 20 kDa was observed. Western blot analysis with His_6_-tag antibodies showed the absence of His_6_-tag in the low-mass fraction (Figure A1). Mass spectra analysis showed the N-terminal fragments of Dnmt3a-CD in the low-mass fraction (data not shown). No further studies have been conducted with this mutant.

**Figure 3:**
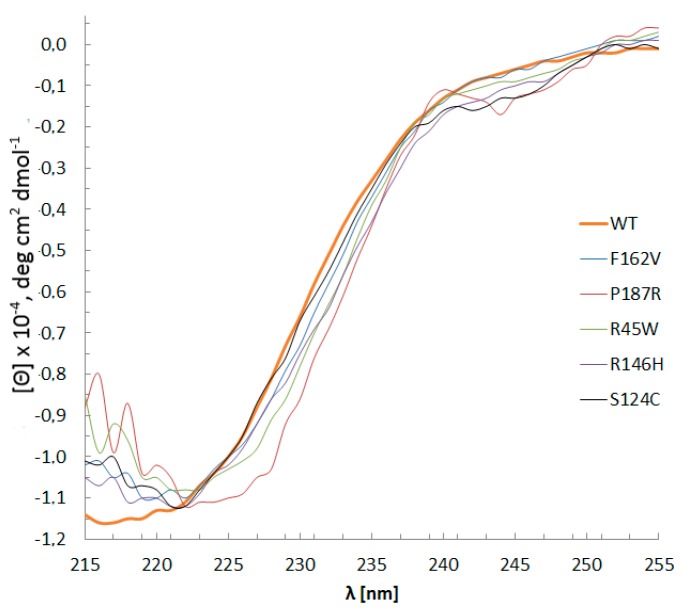
WT Dnmt3a-CD and mutants CD spectra. Buffer A with 0.1 mM AdoHcy, path length 0.5 mm, 15 °C, protein concentration 0.1–0.4 g/L. Data represents the averaged results from three measurements.

The CD spectra of WT Dnmt3a-CD and mutant enzymes (Figure 3) did not reveal remarkable changes in the secondary structure distribution. This result indicates that all the mutant proteins were properly folded. Judging by the ellipticity values at 222 nm, the α-helical content in the mutants, apparently, had not changed. Small differences were observed only in the case of the P187R, which may indicate a slight disturbance of the α-helix percent.

### 3.2. Effect of Cancer-Associated Mutations of Dnmt3a-CD on DNA Methylation

**Figure 4:**
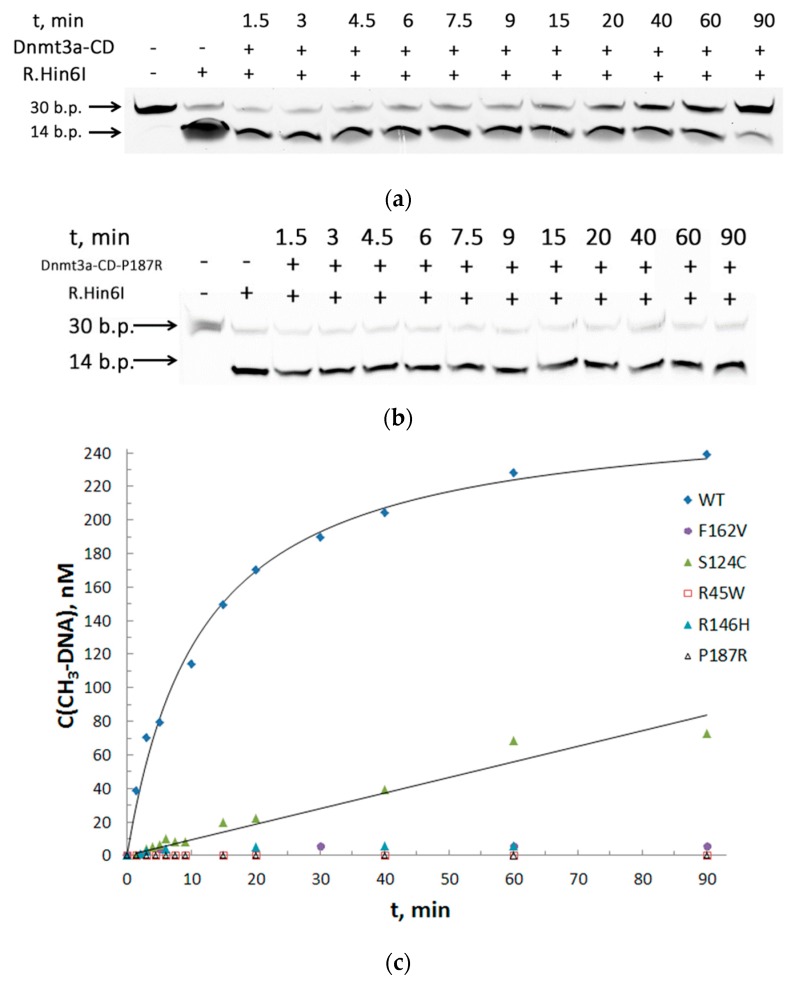
Effect of cancer-associated mutations of Dnmt3a-CD on DNA methylation. (**a**,**b**) Cleavage of 0.3 μM fCG/GCf with Hin6I endonuclease after its methylation with 2 μM WT Dnmt3a-CD or P187R; 20% PAG with 7 M urea; (**c**) Time courses of methylation of fCG/GCf by WT and mutant Dnmt3a-CD. [(CH_3_)-DNA] values were calculated based on the data from (**a**) and (**b**) (see Materials and Methods). t represents the time of methylation reaction.

To assess the effect of each mutation on DNA methylation, we used a one-site, 30 bp CpG-containing DNA substrate fCG/GCf (Table 2). The methylation activity of Dnmt3a-CD was measured by the protection of methylated DNA from cleavage by restriction endonuclease Hin6I [32]. This allowed us to avoid measuring the amount of tritium incorporated into oligonucleotide duplexes used in traditional methylation assay [31]. Thirty base pairs fCG/GCf were cleaved with the formation of 14 bp fluorescent products, the reaction mixtures were analyzed in 20% PAG with 7 M urea (Figure 4A,B). The time courses of methylation of the DNA substrate fCG/GCf by WT and mutant Dnmt3a-CD were obtained (Figure 4C). The curves were linear until 5 and 10 min for WT Dnmt3a-CD and mutants, respectively. In the case of R45W, R146H, and F162V, methylation activity was abolished. The initial rates of methylation (*v_0_*) were determined (Table 3). The efficiency of methylation (*v_0_*^rel^, Table 3) for S124C and P187R was 2.6- and 14-fold, respectively, lower than for the WT Dnmt3a-CD.

**Figure 5:**
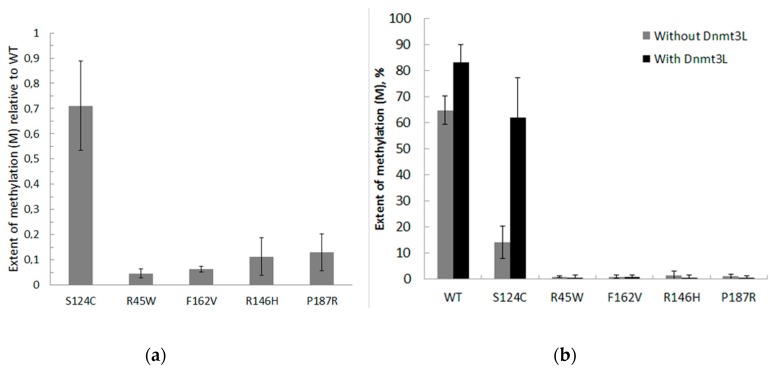
Methylation by WT and mutant Dnmt3a-CD in the presence or absence of Dnmt3L during 2 h. (**a**) Reaction mixtures contained 0.3 μM fCG/GCf DNA substrate, 2 μM Dnmt3a-CD or mutant, and 25 μM AdoMet; data represent the averaged results from at least six independent experiments; (**b**) Reaction mixtures contained 120 nM fCG/GCf DNA substrate, 1 μM WT or mutant Dnmt3a-CD, and 1 μM Dnmt3L; data represent the averaged results from three independent experiments.

Further, to examine the methylation activities for the low activity mutants, the total time of the reaction was increased up to 2 h, the other conditions being the same. The extent of methylation (*M*) for each enzyme relative to WT was determined (Figure 5A). The *M* values for S124C, R181L, and P187R were 1.6-, 2-, 20-fold lower, respectively, compared to the WT Dnmt3a-CD. R45W, R146H, and F162V showed practically no methylation activity.

The ability of enzymatically inactive regulatory factor Dnmt3L to activate WT and mutant Dnmt3a-CD was examined (Figure 5B). Dnmt3a-CD in complex with Dnmt3L forms a linear heterotetramer consisting of two Dnmt3a-CD and two Dnmt3L subunits [34]. Dnmt3L positions the Dnmt3a catalytic loop and improves the catalysis [3]. The Dnmt3L caused a 1.3–4.2-fold increase in WT and S124C methylation activity and did not activate P187R and enzymatically inactive mutants R146H, F162V, and R45W.

Our results allow us to highlight mutations that lead to a decrease (S124C and P187R) or loss (R146H, F162V, and R45W) of methylation activity. To understand the mechanism underlying these phenomena, several steps of methylation reaction were investigated. The DNA methylation reaction mechanism included the following steps: DNA recognition and binding, target cytosine flipping out of the double helix, attack of the conserved cysteine at C6 position and covalent intermediate formation, methyl group transfer from the donor AdoMet, followed by resolution of the intermediate and release of the products [3,35]. Then the binding step and the formation of the covalent intermediate were analyzed in detail.

### 3.3. DNA Binding

**Figure 6:**
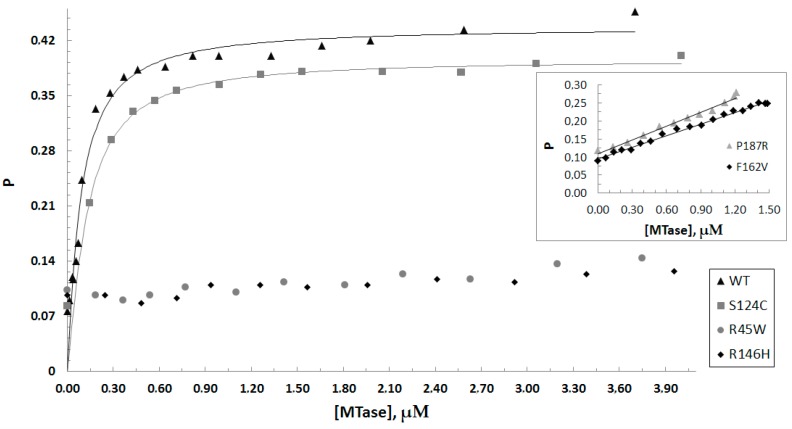
Binding curves for Dnmt3a-CD WT and mutants obtained upon titration of the fCG/CG DNA substrate (10 nM) in the presence of 0.1 mM AdoHcy. P represents the fluorescence polarization. The inset shows binding curves for P187R and F162V on a different scale. Data represent the averaged results from at least two independent experiments.

Dnmt3a-CD/DNA complex formation was monitored by fluorescence polarization of FAM-labeled DNA using a one-site DNA substrate fCG/CG (Table 2). Binding measurements were performed in the presence of the co-factor analog AdoHcy, which mimics the experimental conditions of the methylation assays [36]. For WT and S124C, nearly hyperbolic binding curves were obtained (Figure 6). For P187R and F162V, no plateau on the curves was observed due to a low concentration of the purified enzymes (Figure 6, insert). Surprisingly, R45W and R146H showed no binding to DNA. All the data obtained were analyzed according to the Hill model with a 3-parameter Hill equation considering the cooperative nature of binding of Dnmt3a-CD to DNA [37]. The K_d_ value for the WT was 77 ± 6 nM; the K_d_ values for S124C and F162V were 1.6–3-fold higher relative to the WT (Table 3). The K_d_ value for P187R was not determined. However, the enzyme retained the weak ability to bind DNA.

Overall, the S124C, F162V, and P187R mutations did not affect the ability of Dnmt3a to bind DNA, but R45W and R146H mutations lead to a complete loss of the DNA binding affinity.

### 3.4. Formation of Covalent Intermediates between Dnmt3a-CD and DNA Duplexes Containing 2-Pyrimidinone

**Figure 7:**
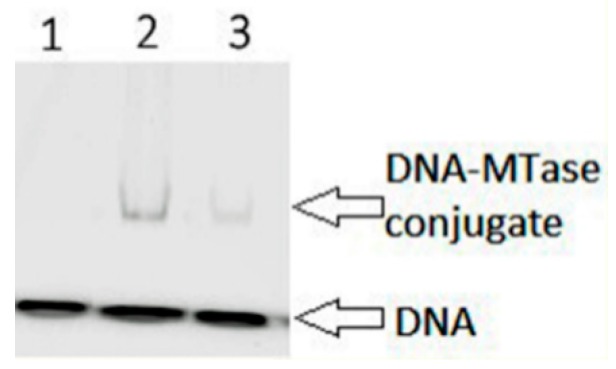
Analysis of the covalent complex formation of WT Dnmt3a-CD and mutants with 2-pyrimidinone-substituted DNA duplex fCG/GZ. Six micromolar WT Dnmt3a-CD or S124C, 0.2 μM fCG/GZ DNA substrate, 0.1 mM AdoHcy. Twelve percent PAG with 0.1% SDS. Lane 1 represents fCG/GZ, lanes 2 and 3 represent fCG/GZ with WT or S124C, respectively.

One of the key steps of the DNA methylation reaction is the formation of the transient covalent complex between the enzyme and its DNA substrate [37]. The FAM-labeled one-site 18 bp DNA substrate fCG/GZ containing 2-pyrimidinone (Z) in the place of the target cytosine was used (Table 2). The nucleophilic attack by the highly conserved cysteine of the motif IV at the C6 position of the cytosine ring produces the covalent conjugate, which triggers the subsequent methyl group transfer from AdoMet to the C5 carbon atom [37]. The covalent complex is resolved by deprotonation at C5 leading to β-elimination of the cysteine residue [3]. Conjugates of C5-MTases with DNA containing Z instead of the target cytosine are stable due to the retardation of β-elimination and can be detected by gel electrophoresis [38]. The ability of WT Dnmt3a-CD and mutants to form a covalent conjugate was analyzed in the presence of co-factor AdoHcy. The covalent conjugate formation was observed only for S124C (Figure 7) but not for the P187R and the F162V (data not shown).

One can conclude that the mutations can be divided into several groups. The first group includes only the S124C mutation, which led to a significant decrease in Dnmt3a-CD methylation activity. The second group consists of mutations, such as F162V and P187R, which reduced methylation activity even in the presence of Dnmt3L but do not affect the ability of enzymes to bind DNA. Moreover, mutations that led to a drastic change in Dnmt3a-CD functioning in all aspects (R45W and R146H) form the third category. The results obtained indicated that all the substituted amino acid residues are important for the proper Dnmt3a-CD functioning.

## 4. Discussion

**Figure 8:**
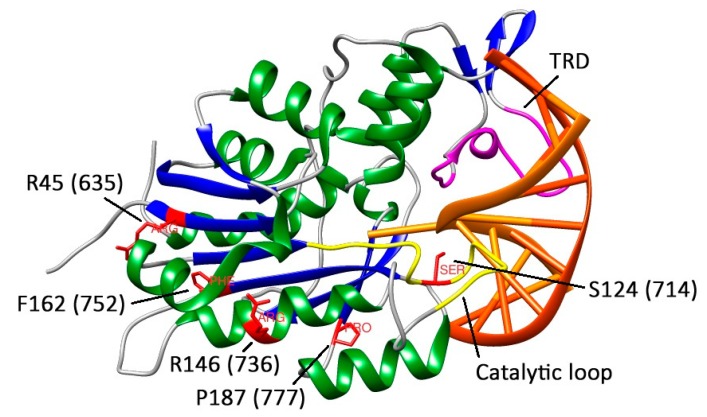
The fragment of DNA-(DNMT3A-CD)-DNMT3L complex structure (PDB: 6F57). DNA is given in orange sticks, DNMT3A-CD is colored by secondary structure (α-helix in green, β-sheet in blue, loops in white), the catalytic loop is colored in yellow, target recognition domain (TRD) is colored in magenta. Residues of interest within human DNMT3A-CD are numbered in accordance with murine Dnmt3a-CD and given in red.

To determine the functional consequences of missense mutations in DNMT3A, we selected six mutations in the catalytic domain of murine Dnmt3a: S124C, R45W, R146H, R181L, P187R, and F162V. Figure 8 highlights the positions of the mutated amino acid residues in the structures of Dnmt3a-CD [25] and DNA-(DNMT3A)-DNMT3L complex (PDB code: 6F57) [30].

S124 (714) is one of the highly conserved amino acids of the motif IV (Figure 2), which is located in the catalytic loop (Figure 8) [30]. Replacement of S124 with cysteine reduced the activity of Dnmt3a-CD (*v_0_*^rel^ value decreased threefold), but only slightly affected the DNA binding affinity (K_d_ increased 1.6-fold). Our data are in accordance with recent studies showing the decreased enzyme activity of the human DNMT3A S714C mutant [29,30]. In human DNMT3A, the S714 residue participates in the formation of electrostatic interactions between the sugar-phosphate backbone and MTase [30]. We suggest that S124 acts as a “hinge” that facilitates the flipping of target cytosine residue out of the DNA helix. Thus, we analyzed the formation of the transient covalent complex between S124C and DNA containing 2-pyrimidinone in place of the target cytosine (fCG/GZ). Consistent with our predictions, the complex was less prominent compared to that of WT (Figure 7). This step is supposed to be coupled with the flipping of the target cytosine [39]. Along with that, S124C was significantly stimulated by activator Dnmt3L (Table 3). Collectively, the negative effect of S124C mutation can be considered moderate.

The R45 (635) is located within the conserved motif I predicted for AdoMet binding (Figure 2) and far away from the Dnmt3a/3L interface [3] (Figure 8). Replacing R45 with tryptophan led to a complete loss of the enzyme activity (Table 3). Similarly, the R635G DNMT3A demonstrated the decrease in methylation activity on the poly dI-dC substrate [29]. In the case of R45W, we observed a complete loss of DNA binding affinity and a lack of activation by Dnmt3L. It is possible that R45W might affect protein structure and stability.

Even though R146H (736), F162V (752), and P187R (777) residues are not the highly conserved across the functional motives I-X of Dnmt3a (Figure 2), we observed a strong impact of these mutations on the methylation reaction (Table 3).

Replacing R146 with histidine led to a complete loss of Dnmt3a-CD methylation activity accompanied by the loss of DNA binding affinity and the absence of activation by Dnmt3L (Table 3). Our data are in accordance with a report demonstrating that the R736A mutation leads to the 20-fold decrease in methylation activity [28]. Recent studies on human R736H DNMT3A have shown the hypermethylation of long multi-CpG DNA substrate (749 bp) and the decrease in methylation activity on the poly dI-dC substrate [29]. Based on these findings, we can suggest the dependence of R146H methylation activity on the DNA substrate. According to the DNA-(DNMT3A)-DNMT3L co-crystal structure, the R146 residue is located within the tetramer interface (Figure 8) [28]. We speculate that R146H mutation disturbed the tetramer interface, similarly to the R882H/C mutation [40], which led to the loss of binding affinity.

Replacement of P187 with arginine and F162 with valine led to a 15-fold decrease or to a complete loss of the methylation activity, respectively (Table 3). Both mutants preserved the ability to bind DNA, but the K_d_ values were at least three times higher than that for the WT. Notably, we did not observe the formation of a transient covalent complex of P187R or F162V with fCG/GZ, and the Dnmt3L activator protein did not increase their methylation activities. The P187 residue is located within the loop connecting the α-helix and the β-sheet (Figure 8) in the hydrophobic core, which consists of aliphatic (Val, Ile, Met) and aromatic (Tyr) amino acids and is located near the positively charged Lys residue. Due to its cyclic structure, proline bestows local rigidity to the enzyme structure, fixing α-helix and β-sheet positions relative to each other. Replacement of proline with arginine may lead to a conflict between the environment and the charged arginine residue. At the same time, this replacement increases local chain mobility. Together, these changes may result in a critical disruption of the enzyme’s tertiary structure. The F162 residue is located near motif VI (ENV). The F162V mutation likely creates a steric clash that can affect the interaction of the ENV tripeptide with the target cytosine residue and lead to the loss of enzymatic activity.

## 5. Conclusions

We studied the effect of five missense mutations in the human DNMT3A catalytic domain found in AML patients. Considering the dramatic decrease in methylation of mutated Dnmt3a-CD, one can suggest functional impairment of DNMT3A during AML progression. We examined the individual steps of the methylation reaction, which may be disturbed due to the mutations. Surprisingly, in the case of R45W and R146H enzymes, the ability to bind DNA was abolished. We believe that these cancer-associated mutations may result in the change of the methylation patterns. Interestingly, in the case of R45W mutation in the AdoMet binding region, the loss of ability to bind DNA was found. The in vitro results can act as a basis for further in vivo analyses, as they are useful for understanding the presence of the aberrant methylation pattern characteristic for AML [17]. Mutated variants of DNMT3A can act as new biomarkers important for developing effective individual therapy and may become attractive targets for personalized medicine.

## Figures and Tables

**Table 1:** Frequency of different Dnmt3a-CD mutations occurred in acute myeloid leukemia (AML) based on the research from OncoKB (Precision Oncology Knowledge Base), CBioPortal (The cBioPortal for Cancer Genomics), TCGA (The Cancer Genome Atlas), and COSMIC (Catalogue of Somatic Mutations In Cancer) databases and [12,19,20,21,22,23,24,25,26,27]. The selected amino acids are given in bold.

Amino Acid Number in Dnmt3a-CD	Amino Acid Number in Full-Length Human DNMT3A	The Number of Patients	The Number of COSMIC References	Allele Frequency
**R45**	**R635W/Q**	**5**	**4**	**0.24**
V46	V636M	2	2	-
**S124**	**S714C**	**6**	**12**	**0.29**
P128	P718L	1	1	-
R139	R729W/Q	3	5	0.11
Y145	Y735C	2	6	-
**R146**	**R736H/C/A/P**	**10**	**10**	**0.49**
**F162**	**F752V**	**2**	**2**	**-**
**R181**	**R771Q/L**	**3**	**3**	**0.42**
**P187**	**P777R**	**2**	**1**	**-**
B191	D781G	1	1	0.1
R202	R792H/C	2	2	0.41
K213	R803S	1	1	-
K251	K841Q	1	1	-
R292	R882H/C/P	173	619	0.28
A319	F909C	1	1	0.17

**Table 2:** DNA duplexes.

Designation	Sequence *
fCG/GCf	5′- FAM - CTGAATACTACTTGC**G**CTCTCTAACCTGAT 3′- GACTTATGATGAAC**G**CGAGAGATTGGACTA - FAM
fCG/CG	5′- FAM - CTGAATACTACTTGC**G**CTCTCTAACCTGAT 3′- GACTTATGATGAAC**G**CGAGAGATTGGACTA
fCG/GZ	5′- FAM - GAGCCAAGC**G**CACTCTGA 3′- CTCGGTTC**G***Z*GTGAGACT
fCG/GC	5′- FAM - GAGCCAAGC**G**CACTCTGA 3′ - CTCGGTTC**G**CGTGAGACT
CGZ/GCf	5′ - GAGCCAAGC**G***Z*ACTCTGA 3′ - CTCGGTTC**G**CGTGAGACT - FAM

* FAM, f, 6-carboxyfluorescein; Z (2-pyrimidinone) and CG dinucleotides are written in bold. Target cytosine residues are underlined.

**Table 3:** Methylation activity, DNA binding affinity, Dnmt3L activation, and covalent complex formation with 2-pyrimidinone-substituted DNA duplex fCG/GZ by WT and mutant Dnmt3a-CD. Data represent the results from at least three independent experiments.

Exchange	*v_0_*, nM/min **	*v_0_*^rel^, %	K_d_, nM	K_d_^rel^	Ability of Dnmt3L to Enhance Dnmt3a Functionality	Covalent Complex Formation with fCG/GZ
WT	15.7 ± 1	100	77 ± 6	1.00	Yes	Yes
S124C	6.2 ± 4	39	127 ± 44	1.63	Yes	Yes
R146H	No activity	No activity	No binding	No binding	No	n.d.*
R45W						
F162V			232 ± 32	3.01	No	No
P187R	1.1 ± 1	7	n.d. *	n.d. *	No	No

* n.d.–not determined, ** the same concentration of WT or mutant Dnmt3a was used in each experiment.
